# Analysis of the Mercury Distribution in Blood as a Potential Tool for Exposure Assessment — Results from Two Artisanal and Small-Scale Gold Mining Areas in Zimbabwe

**DOI:** 10.1007/s12011-021-02714-1

**Published:** 2021-04-23

**Authors:** Anna-Maria Wahl, Stephan Bose-O’Reilly, Viola Mambrey, James P. K. Rooney, Dennis Shoko, Dingani Moyo, Shamiso Muteti-Fana, Nadine Steckling-Muschack, Stefan Rakete

**Affiliations:** 1 Institute and Clinic for Occupational, Social and Environmental Medicine, University Hospital, LMU Munich, Ziemssenstr. 1, D-80336 Munich, Germany; 2 Institute of Public Health, Medical Decision Making and Health Technology Assessment, Department of Public Health, Health Services Research and Health Technology Assessment, UMIT (Private University for Health Sciences, Medical Informatics and Technology), Hall in Tirol, Austria; 3 University Children’s Hospital Regensburg (KUNO-Clinics), University of Regensburg, Clinic St. Hedwig, Regensburg, Germany; 4 Academic Unit of Neurology, Trinity Biomedical Sciences Institute, Trinity College Dublin, Dublin, Ireland; 5 Tailjet Consultancy Services, Harare, Zimbabwe; 6 School of Public Health, Faculty of Health Sciences, Occupational Health Division, University of the Witwatersrand, Johannesburg, South Africa; 7 Faculty of Medicine and Faculty of Social Sciences, Midlands State University, Gweru, Zimbabwe; 8 Department of Community Medicine, UZ College of Health Sciences, Harare, Zimbabwe

**Keywords:** Artisanal and small-scale gold mining, ASGM, Zimbabwe, Exposure assessment, Mercury, Human biomonitoring

## Abstract

People in artisanal and small-scale gold mining (ASGM) areas are frequently exposed to high levels of mercury (Hg). Hg analyses in urine and whole blood are the gold standard of biomonitoring, although this may not provide sufficient information about the source of exposure, e.g., due to the use of Hg for gold extraction or due to nutrition. To evaluate, whether the pharmacokinetic properties of individual Hg species may be useful for exposure assessment, we determined the Hg levels in different blood components from 199 participants. Therefore, whole blood was centrifuged on-site to yield erythrocytes and plasma. Globin was isolated from the erythrocytes by precipitation with ethyl acetate. Albumin was isolated from plasma by gradual precipitation with saturated ammonium sulfate solution. Hg levels in all samples were determined by using a direct Hg analyzer. Median Hg levels for whole blood, erythrocytes, and plasma were 2.7, 3.7, and 1.3 μg/l, respectively. In globin and albumin, median Hg levels were 10.3 and 7.9 μg/kg, respectively. The distribution of Hg was strongly correlated with whole blood Hg levels (*p* < 0.01) and the time between the last use of Hg and the date of the participation (*p* < 0.01). The results suggest that the distribution of Hg in blood is substantially affected by the extent and the frequency of the exposure to elemental Hg. Therefore, the analysis of Hg in erythrocytes and plasma may be a valuable tool for Hg exposure assessment in ASGM areas.

**Supplementary Information:**

The online version contains supplementary material available at 10.1007/s12011-021-02714-1.

## Introduction

Despite its international condemnation, mercury (Hg) is still heavily used in artisanal and small-scale gold mining (ASGM), which accounts for approximately 38 % of the global annual Hg emissions [1]. Due to its simplicity, amalgamation of gold remains the primary method of choice in ASGM. On a global scale, 10 to 19 million people are involved, threatened by serious health consequences due to acute and chronic Hg exposure [2–5]. The primary exposure pathway in ASGM is the inhalation of elemental Hg, which is primarily released during amalgam smelting. In fact, amalgam smelters had a significantly higher Hg body burden compared to gold panners or the general population living in ASGM areas [1]. In addition to elemental Hg, the dietary uptake of methylmercury (MeHg, secondary exposure) is a serious contributor to Hg exposure, too [6].

In general, biomonitoring studies focus on the analysis of total Hg in whole blood or urine. However, this does not allow the exact determination of the source of exposure, which may require Hg speciation, e.g., by GC-CVAFS [7] or LC-ICP-MS [8]. Due to its strong affinity to sulfhydryl groups in proteins, Hg species can also be analyzed by proteomics [9–11]. Although Hg speciation provides the most accurate information for exposure assessment, all of these methods require sophisticated laboratory infrastructure that is likely not available in an average trace element laboratory. Alternatively, the pharmacokinetic properties of elemental Hg and MeHg may be used to identify the source of exposure. It is known that elemental Hg is almost equally distributed between erythrocytes and plasma, whereas MeHg is predominantly found in the erythrocyte fraction [12]. Consequently, different exposure scenarios may result in distinct distribution patterns of Hg in blood.

Therefore, the main objective of this study was the investigation of Hg distribution patterns in the blood of individuals that identified themselves as artisanal and small-scale gold miners from the districts of Kadoma and Shurugwi in Zimbabwe. This included the analyses of Hg levels in whole blood, erythrocytes, and plasma but also in the major blood proteins globin and albumin. Furthermore, the results were correlated with sociodemographic and exposure information to evaluate if Hg distribution patterns in blood can complement the exposure assessment in ASGM areas by using relatively simple methods.

## Methods and Materials

### Study Population

This study was designed as a cross-sectional epidemiological study. The local partners (DM, DS) selected Kadoma and Shurugwi as representative ASGM areas in Zimbabwe. The study centers were located at two hospitals in Shurugwi and Kadoma to perform the health assessments and specimen sampling. The target population of this study consisted of people that identified themselves as miners and were at least 18 years of age. The local partners established the first contact with the participants and organized the transport of the participants to the study locations. All participants received the participant information to inform them about the study, signed the consent form, and received US $5 as compensation for the lost working day. Participation was voluntary at all times. From March 18th to March 29th, 2019, a total of 207 participants, 134 were from Kadoma and 73 from Shurugwi, were examined, and whole blood samples as well as urine were collected. Additionally, erythrocytes and plasma fractions were collected from 201 participants (for details, see below). Please refer to Mambrey et al. for details regarding the physical examinations and questionnaires [13]. The participants were informed of their results by the principal investigator in Zimbabwe (DM).

### Materials

Ammonium sulfate was obtained from Roth (Karlsruhe, Germany). Isopropanol was obtained from Sigma-Aldrich (St. Louis, USA). Ethyl acetate, sodium chloride, and hydrochloric acid (30%) were obtained from Merck (Darmstadt, Germany). All chemicals were at least of analytical grade.

### Sampling of Whole Blood, Erythrocytes, and Plasma

Trained medical personnel took venous whole blood samples from each participant into a 7 ml Li-heparin tube certified for trace metal analysis (Sarstedt®). The names of the participants were encoded, and the decoding form was kept separately by the principal investigator (D.M.). Whole blood was separated on-site into an erythrocyte and a plasma fraction by centrifugation. Aliquots of erythrocytes and plasma were pipetted into 2-ml cryovials and immediately cooled to 4 °C, transported to Germany and frozen at − 18 °C until further processing. This methodology was used at both study locations and carried out by the same research team.

### Isolation of Globin and Albumin

After the transport of the samples to the laboratory, globin and albumin were isolated from the erythrocytes and plasma, respectively. Each sample was processed at least in duplicate. The isolation of globin and albumin was tested in preliminary experiments prior to this study (unpublished data).

*Globin* — Globin was isolated according to an existing literature protocol with some modifications [14]. In detail, the frozen erythrocytes were thawed at room temperature on a roll mixer. Thereafter, the sample was vortexed, and an aliquot of 0.5 ml was used for globin isolation according to the protocol. After the final step, globin was yielded as a light gray solid and dried overnight in a fume hood after covering the centrifuge tube with cellulose tissue. The resulting white powder was directly analyzed or frozen at − 18 °C until further use.

*Albumin* — Albumin was isolated according to an existing literature protocol with some modifications [15]. In detail, the frozen plasma fraction was thawed at room temperature on a roller mixer. After that, an aliquot of 0.5 ml was pipetted into a 2 ml centrifugation tube, and albumin was isolated by gradual precipitation with a saturated ammonium sulfate solution according to the protocol. After the first two additions of ammonium sulfate, samples were centrifuged for 15 min at 7300 and 13,000 g, respectively, and the resulting supernatants were transferred into new tubes. After the final addition of ammonium sulfate, the sample was centrifuged at 13,000 g for 25 min. The supernatant was discarded, and the precipitate was covered with cellulose tissue and dried overnight in a fume hood. Albumin was yielded as a white-yellowish crystalline powder. The samples were directly analyzed or stored at − 18 °C until further use.

### Mercury Analysis

Total Hg levels were analyzed in erythrocytes, plasma, globin, and albumin by means of direct mercury analysis (DMA-80 evo, MLS-MWS, Leutkirch, Germany). Whole blood samples from the same participants were analyzed in a previous study [13]. For erythrocytes and plasma, 100 μl were directly pipetted into the sample boats. For globin and albumin, between 8 and 25 μg were weighed into the sample boats. All subsequent steps were part of the automated Hg analysis. Hg was detected by atomic absorption at 253.5 nm. Quantitation is based on an external calibration. The detection limit for total Hg in whole blood, erythrocytes, and plasma was 0.05 μg/l using a sample volume of 100 μl. For isolated proteins, the detection limit was 0.5 μg/kg using a sample weight of 10 μg. Aqueous Hg standards (10 μg/l) and two certified reference materials (ClinChek®, Recipe, Munich, Germany) for whole blood (1.26 and 6.87 μg/l) were analyzed each day for internal quality assurance.

### Outcome and Explanatory Variables

For this study, Hg levels in whole blood, erythrocytes, plasma, globin, and albumin were treated as outcome variables. Additionally, the ratio of the Hg levels in erythrocytes and plasma (Hg_E/P_) were calculated and used for statistical analysis. Various factors that are associated with Hg exposure were collected during the field work, including sociodemographic factors, working conditions, and health-related data from the study participants as reported earlier were treated as explanatory variables [13]. In detail, information on age, gender, the last time Hg was used, exposure risk factors, fish consumption, alcohol consumption, malaria disease, and place of residence were used for this study. The risk of a higher exposure to Hg was evaluated through an exposure risk score (ERS), which ranges from 0 to 3, depending on the categories *Mercury Storage, Retort Use and Work Clothes* [13]. The following data were considered as possible confounders for Hg levels in blood: fish consumption, alcohol consumption, and malaria disease.

### Statistical Analysis

For six of the 207 participants, erythrocytes and plasma samples were not collected during the field work. Furthermore, sample volumes from two participants were too low for protein isolation. Consequently, eight participants were excluded from further analysis. The remaining 199 participants had a complete set of Hg values for all five matrices (whole blood, erythrocytes, plasma, globin, and albumin). From this group, one participant was excluded from analysis because of sample contamination. In summary, the data from 198 participants were included in the statistical analysis. SPSS® Statistics (Version 26, IBM Corporation, New York, USA) and R (version 3.6.2.) were used to analyze the data. Continuous variables were summarized using median, interquartile ranges, minimum, and maximum. Categorical and nominal variables were summarized using frequencies and percentages. Due to the non-parametric distribution of Hg levels, Spearman-Rho, Mann-Whitney *U*, Kruskal-Wallis, and Jonckheere-Terpstra tests were applied. Scatter plots, box plots, and density plots were used for visual representation. Finally, linear regression modelling was used to explore associations between log (Hg_E/P_) and exposure factors adjusted for sociodemographic variables. A separate linear model was built for each exposure factor and in each case adjusted for gender, age, place of residence, and fish consumption. A model for time from last Hg contact was built without statistical adjustments due to a higher proportion of missing values for this variable.

### Threshold Values

Threshold values by German Federal Environmental Agency (UBA) were used to stratify the results according to the Hg levels in whole blood [16]. In detail, samples with a whole blood Hg level of either below 5 μg/l (HBM-I), between 5 and 15 μg/l, or above 15 μg/l (HBM-II) were assigned to stratified groups for statistical analysis.

## Results

The sociodemographic data and available exposure factors of the study population are summarized in Table 1 and Table S1. Information on age, gender, living area, and fish consumption were available for all participants included in the analysis. Data for exposure risk, alcohol consumption, and malaria was available for 170 (86%) participants, data on the most recent use of Hg for 88 participants (44 %).

**Table 1:** Demographic details of the study population

Age	N	198	
	Median (Min.–Max.)	38 (18–77)	
		N	%
Gender	Males	162	(81.8)
	Females	36	(18.2)
Living Area	Kadoma	128	(64.6)
	Shurugwi	70	(35.4)
Last time Hg	1–2 days	33	(16.7)
	3 days–4 weeks	38	(19.2)
	> 4 weeks	16	(8.1)
	Missing	111	(56.1)
Exposure risk score	0	20	(10.1)
(Exposure risk factors: Retort use (yes/no). Work clothes at home (no/yes). Hg storage [no (at work/yes (at home)])	1	63	(31.8)
	2	59	(29.8)
	3	28	(14.1)
	Missing	28	(14.1)
Fish consumption	< once a week	41	(20.7)
	> once a week	157	(79.3)

### Mercury Levels in Whole Blood, Erythrocytes, Plasma, Globin, and Albumin

**Fig. 1:**
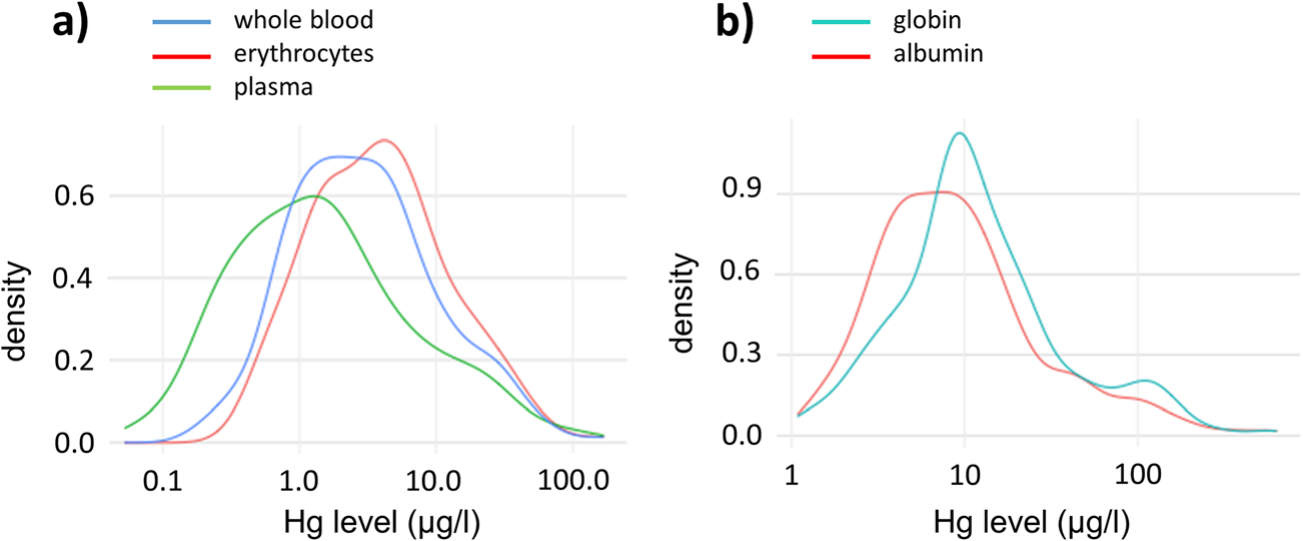
Density plots of the logarithmized Hg levels in liquids (**a**: Hg in whole blood (blue), erythrocytes (red), and plasma (green); results are given in μg/l) and isolated proteins (**b**: Hg in globin (blue) and albumin (red); results are given in μg/kg)

The results of the Hg analyses in the blood components are given in Table 2. According to the threshold levels for Hg in whole blood by the UBA, 133 participants (67%) were below the HBM-I value, 43 (22%) between HBM-I and HBM-II, and 22 (12%) above the HBM-II value. Theoretical whole blood mercury levels calculated from Hg levels in erythrocytes and plasma correlated very well with actual levels of Hg in whole blood (Fig. S1). The Hg levels in all matrices were non-parametrically distributed. Hg levels in erythrocytes were on average higher than in whole blood and plasma (Table 2 and Fig. 1a). Similarly, Hg levels in globin were higher than in plasma (Table 2 and Fig. 1b). The Hg levels in all matrices were highly correlated to each other (r > 0.65, Table S2). Correlations were particularly strong for erythrocytes and globin as well as for plasma and albumin (Fig. S2).

**Table 2:** Results of the mercury analysis in the blood components of all samples (*n* = 198, *GM* geometric mean)

	Whole blood	Erythrocytes	Plasma	Globin	Albumin	Hg_E/P_
	μg/l			μg/kg		
Minimum	0.2	0.3	0.1	1.1	1.3	0.5
25th perc.	1.2	1.6	0.5	6.8	4.2	1.3
Median	2.7	3.8	1.3	10.4	7.9	2.3
75th perc.	6.3	8.0	4.1	21.4	15.8	5.0
Maximum	166.8	164.5	164.9	573.0	631.4	20.8

### Correlations of Hg Levels in Whole Blood with Hg Levels in Erythrocytes, Plasma, Globin, and Albumin

**Fig. 2:**
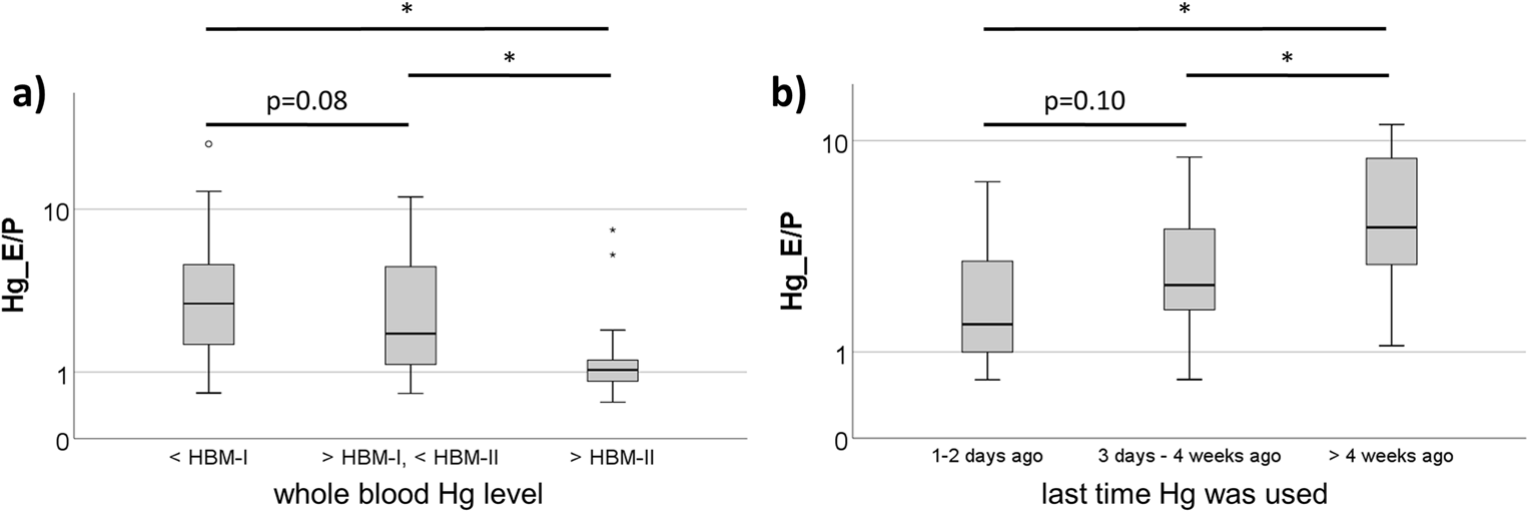
Box plots of the Hg distribution in whole blood expressed by the ratio of Hg levels in erythrocytes and plasma (Hg_E/P_) in relation to the Hg levels in whole blood (**a**, grouped by HBM categories) and the last time mercury was used (**b**). *p < 0.01 (Kruskal-Wallis test)

Table 3 shows that when outcomes were stratified by HBM values, median Hg levels for all matrices increased with higher HBM categories. With increasing whole blood Hg levels, a significantly higher percentage of Hg was found in plasma, resulting in a significantly lower Hg_E/P_ in samples with whole blood Hg levels above HBM-II when compared to the other groups (Fig. 2a).

**Table 3:** Stratification of Hg levels in erythrocytes, plasma, globin, and albumin by whole blood Hg levels (HBM categories)

	*n*	Whole blood	Erythrocytes	Plasma	Globin	Albumin	Hg_E/P_
		[μg/l]			[μg/kg]		
< HBM-I	133	1.6	2.1	0.7	8.0	5.4	3.1
> HBM-I, < HBM-II	43	6.6	9.5	4.8	22.0	17.4	2.0
> HBM-II	22	27.1	28.9	24.0	112.6	87.8	1.0
p*		< 0.001	< 0.001	< 0.001	< 0.001	< 0.001	< 0.001

*Jonckheere-Terpstra test

All Hg values are given as medians.

### Correlation of Recent Mercury Exposure and Exposure Risk Factors on Mercury Levels in Erythrocytes, Plasma, Globin, and Albumin

The Hg levels in all matrices except for erythrocytes significantly decreased with the time since last Hg use (Table 4). In contrast, Hg_E/P_ increased from the time of last Hg use (Fig. 2b). For exposure assessment, an exposure risk score (ERS) was used, with 0 being the lowest exposure risk and 3 the highest [13]. The Hg levels in all matrices were mainly positively correlated with an increasing number of risk factors (Table S3). However, no association between ERS and Hg_E/P_ was observed (Fig. S3).

**Table 4:** Stratification of Hg levels in erythrocytes, plasma, globin, and albumin by the time since the last use of Hg

	n	Whole blood	Erythrocytes	Plasma	Globin	Albumin	Hg_E/P_
		[μg/l]			[μg/kg]		
1–2 days	33	5.1	5.8	2.9	16.5	13.5	1.5
3 days–4 weeks	38	2.5	3.4	1.5	11.9	8.8	2.4
> 4 weeks	16	2.0	3.5	0.6	8.5	4.5	4.5
p*		0.017	0.029	0.001	0.013	0.001	0.001

*Jonckheere-Terpstra test

All Hg values are given as medians.

### Correlation of Other Factors with Mercury Levels in Whole Blood, Erythrocytes, Plasma, Globin, and Albumin

The Hg levels in whole blood and individual blood components were stratified by gender, fish consumption, alcohol consumption, and malaria disease (Table S4). Women had significantly higher Hg levels in all matrices, except for albumin. Hg_E/P_ was higher in women. However, the difference was not significant. Hg levels in all matrices were higher for participants that at least ate fish once a week, though a significant difference was only observed for erythrocytes. Although the Hg_E/P_ in this group was higher, too, the difference was not significant. For alcohol consumption and malaria disease, no significant differences were found.

### Linear Regression Modelling

Results from multivariable linear regression models are shown in Table 5. Only never storing Hg at home was significantly associated with the log (Hg_E/P_) ratio with a parameter estimate of 0.50 (95% CI, 0.01–0.99; *p* = 0.044) after adjustment for gender, age, place of residence, and frequency of fish consumption. In the separate univariable model with the last time of Hg exposure as the explanatory variable and log(Hg_E/P_) as the outcome, having a last time from Hg exposure of over 4 weeks relative to 1 to 2 days was significantly associated with an increased log(Hg_E/P_) with a parameter estimate 0.879 (95% CI, 0.436 to 1.322; *p* < 0.001). However, a last time from Hg exposure of 3 days to 4 weeks prior to the examination was associated with a higher ratio, but this parameter did not meet significance with an estimate of 0.312 (95% CI, − 0.034, 0.658; *p* = 0.077).

**Table 5:** Adjusted linear regression estimates of exposure risks vs log of the ratio of Hg in erythrocytes and plasma (Hg_E/P_)

Variable¶	Estimate	2.5% CI	95% CI	*p* value
Retort use: yes	0.02	− 0.31	0.36	0.888
Work clothes at home: yes	− 0.12	− 0.39	0.14	0.363
Hg storage: at work	− 0.09	− 0.37	0.18	0.512
Hg storage: never	0.50	0.01	0.99	0.044

^¶^All estimates adjusted for age, gender, district (Shurugwi or Kadoma), and fish consumption frequency

## Discussion

This cross-sectional study aimed to identify differences in the distribution of Hg levels between multiple blood components in participants identifying themselves as miners from two ASGM areas in Zimbabwe. In fact, the distribution of Hg in blood correlated with exposure factors such as the last time Hg was used at work. Furthermore, a linear relationship was observed between liquid blood components and their individual proteins.

Although the Hg levels in whole blood were lower than what has been found in other ASGM studies, they were still considerably higher than what can be expected in the general population [17,18, 19, 20]. In fact, one third of the participants were above the HBM-I value, which we used as threshold value. However, Hg levels in erythrocytes, plasma, globin, and albumin have never been analyzed thus far in individuals living and working in ASGM areas. Therefore, comparison of these values with other studies was not possible. Hg levels in globin and albumin were primarily analyzed to evaluate, if these proteins can be used for further investigations, e.g., for proteomic analysis. Although artifacts and loss of Hg during the isolation process cannot be excluded, we found a very strong linear relationship for erythrocytes and globin as well as for plasma and albumin. This indicates that the isolated proteins indeed resemble the Hg levels in erythrocytes and plasma, respectively.

In general, Hg levels in all matrices were positively correlated with whole blood Hg levels, indicating that the exposure to Hg causes a ubiquitous increase of Hg levels in blood. However, this increase is not uniform. The distribution significantly correlated with whole blood Hg levels and the last time Hg has been used. An equal distribution between erythrocytes and plasma (Hg_E/P_ = 1) indicates that these participants have likely been recently exposed to mainly elemental Hg. Especially individuals with whole blood Hg levels above HBM-II showed a low Hg_E/P_, probably due to severe exposure to elemental Hg, e.g., by smelting amalgam [18, 19]. Nevertheless, low Hg_E/P_ were also observed for participants with low whole blood Hg levels. After multivariable regression analysis, only never storing mercury at home was significantly associated with higher log (Hg_E/P_). Although higher Hg_E/P_ were also observed for those who eat fish at least once a week, this difference was not significant, and we had adjusted for fish consumption in regression models. Unfortunately, Hg levels in regional fish or other food were not available for a more precise assessment. Similarly, participants with low whole blood Hg levels and that have not been using Hg for more than 4 weeks showed a relatively high Hg_E/P_. This is in keeping with the shorter half-life of Hg in plasma compared to erythrocytes previously described in the literature [12]. Therefore Hg_E/P_ will likely increase with time after the exposure event. However, Hg_E/P_ may also be influenced by other sources of exposure as well as sociodemographic and genetic factors, too. Unfortunately, this data was unavailable, and the number of participants was too low to further explore the impact of these factors.

## Strengths and Limitations

Limitations regarding the participants of this study were already addressed by Mambrey et al. [13]. In short, the poor economic situation and lack of prospects in Zimbabwe is a major reason why people are securing their livelihoods with ASGM. This leads to many illegal and poorly trained workers in gold mining. The participants may also self-identify as miners in order to obtain $5 as compensation, and there was no possibility for the examiners to verify this information. Another general problem, not only concerning this study, was the fact that the individual miners have very different levels of exposure to elemental Hg. Some workers might have been continuously exposed and others just periodically [3]. In the end, exact exposure data was not available. Furthermore, speciation of Hg in the blood samples was not possible due to technical and financial limitations. This would have help to make assumptions about the source of exposure and help to explain distribution patterns of Hg in blood.

Additionally, the quality of the questionnaire data must be scrutinized regarding exposure and socioeconomic data. There is likely a recall bias, especially regarding the last time Hg was used and fish consumption, but also for exposure risk factors and others. This strongly influences the quality of the statistical analysis. Further limitations result from the protein isolation methods, which were not designed for Hg analysis. Consequently, the loss of Hg during the work-up and formations of artifacts cannot be completely excluded. To overcome this, we tested the isolation of proteins in preliminary experiments prior to this study and could not observe significant losses or artifact formation during the work-up.

The strength of the study is the use of relatively simple methods for the assessment of the source, and time of exposure to elemental Hg is one of the strengths of this study, which only requires the separation of whole blood and individual analysis of in erythrocytes and plasma.

## Conclusion

This is, to the best of our knowledge, the first study that addresses the distribution of Hg levels in erythrocytes and plasma as a potential tool for the evaluation of sources of Hg exposure. Despite the uncertainties regarding the participant’s questionnaire data, we were still able to identify Hg distribution patterns in blood depending on the exposure to elemental Hg. In fact, the ratio of Hg levels in erythrocytes and plasma (Hg_E/P_) was associated with recent exposure to elemental Hg. Furthermore, this study showed that the isolation of globin and albumin from blood samples can be used for a more comprehensive analysis of Hg adducts, e.g., by mass spectrometry. In summary, the analysis of Hg in different blood components can provide useful information for Hg exposure assessment.

## Supplementary Information

ESM 1 (PDF 234 kb)

## Data Availability

Individual participant data will not be made available. Anonymized data may be made available upon reasonable request.
