# Supplementary material for: Analysis of the Mercury Distribution in Blood as a Potential Tool for Exposure Assessment — Results from Two Artisanal and Small-Scale Gold Mining Areas in Zimbabwe
Source: Biol Trace Elem Res. 2021 Apr 23;200(3):961–8. doi: 10.1007/s12011-021-02714-1 (PMC8761143; doi:10.1007/s12011-021-02714-1)
Supplement: Supplementary file 1 — (PDF 234 kb) [file 12011_2021_2714_MOESM1_ESM.pdf]

Supplementary information:

The supplementary information provides additional data on the influence of exposure risk factors and potential confounders on the distribution of Hg in blood. Furthermore, it shows the detailed correlation of Hg levels in erythrocytes and plasma with its corresponding proteins globin and albumin.

Table S1: Additional details of the study population (alcohol consumption and malaria disease).

		N	(%)
**Alcohol consumption**	Never	69	(34.8)
	> once a month	14	(7.1)
	> once a week	42	(21.2)
	> once a day	45	(22.7)
	Missing	28	(14.1)
**Malaria**	No	91	(46.0)
	Yes	79	(39.9)
	Missing	28	(14.1)

Table S2: Correlations of Hg levels in the investigated matrices (n=198, Spearman-Rho)

Matrix	erythrocytes	plasma	globin	albumin
**whole blood**	0.97	0.89	0.85	0.69
**erythrocytes**		0.84	0.81	0.65
**plasma**			0.79	0.79
**globin**				0.65

Table S3: Stratification of Hg levels in whole blood, erythrocytes, plasma, globin and albumin by the Exposure Risk Score (ERS).

ERS	n	whole blood [µg/l]	erythrocytes [µg/l]	plasma [µg/l]	globin [µg/kg]	albumin [µg/l]	$Hg_{E/P}$
**0**	20	0.8	1.3	0.5	6.0	5.2	2.3
**1**	63	2.1	2.7	1.2	9.8	6.8	2.3
**2**	59	2.5	3.4	1.1	11.5	7.2	2.5
**3**	28	4.0	4.9	1.7	10.2	9.3	3.0
**p***		0.002	0.001	0.006	0.108	0.162	0.764

* Jonckheera-Terpstra-Test. All Hg values are given as medians.

Table S4: Hg levels in whole blood, erythrocytes, plasma, globin and albumin stratified by gender (a), fish consumption (b), alcohol consumption (c) and malaria disease (d).

a) gender	n	whole blood [µg/l]	erythrocytes [µg/l]	plasma [µg/l]	globin [µg/kg]	albumin [µg/l]	$Hg_{E/P}$
**women**	36	4.7	8.1	1.7	13.1	8.9	3.7
**men**	162	2.4	3.2	1.2	9.5	7.8	2.2
**p#**		0.002	< 0.001	0.049	0.015	0.436	0.068

b) fish consumption	n	whole blood [µg/l]	erythrocytes [µg/l]	plasma [µg/l]	globin [µg/kg]	albumin [µg/l]	$Hg_{E/P}$
**never/ less than once a week**	41	2.2	2.3	1.1	8.3	7.2	1.9
**more once a week**	157	3.1	4.3	1.4	11.2	8.2	2.4
**p#**		0.125	0.026	0.397	0.183	0.408	0.084

c) alcohol consumption	n	whole blood [µg/l]	erythrocytes [µg/l]	plasma [µg/l]	globin [µg/kg]	albumin [µg/l]	$Hg_{E/P}$
**never**	69	2.0	2.9	0.8	9.0	6.5	2.4
**at least once a month**	14	3.3	5.0	1.5	12.8	6.8	2.9
**at least once a week**	42	2.2	2.8	0.9	10.8	6.4	2.6
**daily**	45	3.4	4.0	2.6	10.3	11.3	1.9
**p***		0.062	0.149	0.058	0.163	0.068	0.294

d) Malaria disease	n	whole blood [µg/l]	erythrocytes [µg/l]	plasma [µg/l]	globin [µg/kg]	albumin [µg/l]	$Hg_{E/P}$
**no**	91	2.3	3.1	1.1	11.3	6.7	2.7
**yes**	79	2.9	3.6	1.4	9.4	8.8	2.0
**p#**		0.422	0.533	0.269	0.312	0.151	0.211

Differences between the groups were tested by # Mann-Whitney-U-Test and * Jonckheere-Tepstra-Test. All Hg values are given as medians.

Figure S1: Correlation of analyzed Hg levels in whole blood vs. calculated Hg levels in whole blood. Theoretical Hg levels were calculated from the analyzed Hg levels in erythrocytes (Ery) and plasma (Pla) using the average distribution factor of erythrocytes and plasma in whole blood (n=198, Spearman-Rho, r=0.98). Hg levels in whole blood were calculated as follow: Females: Hg_{WB} = 0.42*Hg_{Ery} + 0.58*Hg_{Pla}; Males: Hg_{WB} = 0.45*Hg_{Ery} + 0.55*Hg_{Pla}. The dotted line is the identity line.

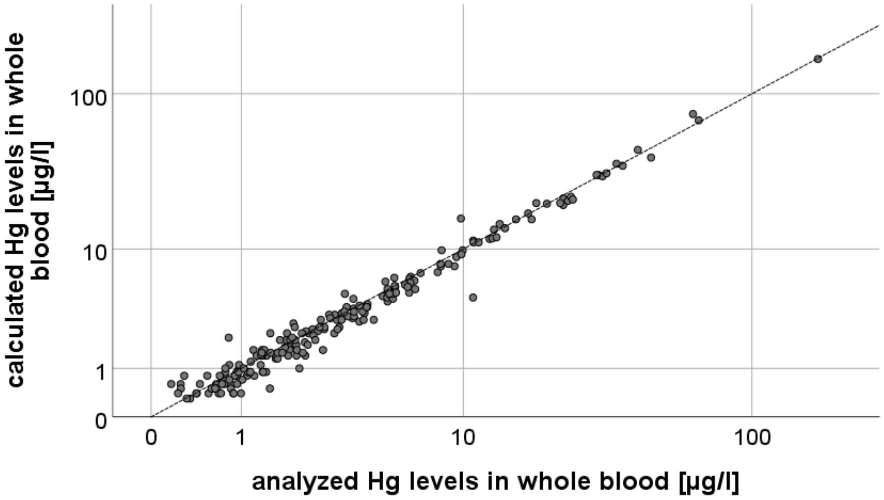

Figure S2: Scatter plot of Hg levels in erythrocytes vs globin (a: n=198, Spearman-Rho, r=0.81) and plasma vs albumin (b: n=198, Spearman-Rho, r=0.80).

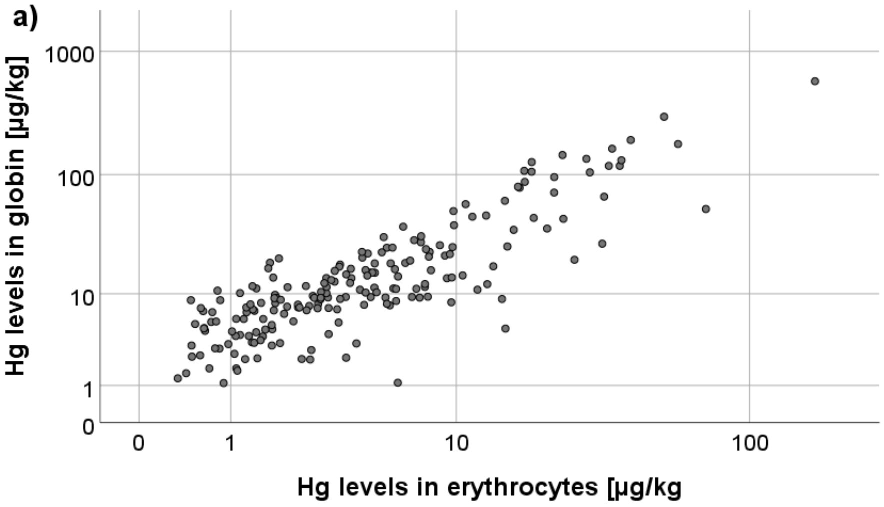

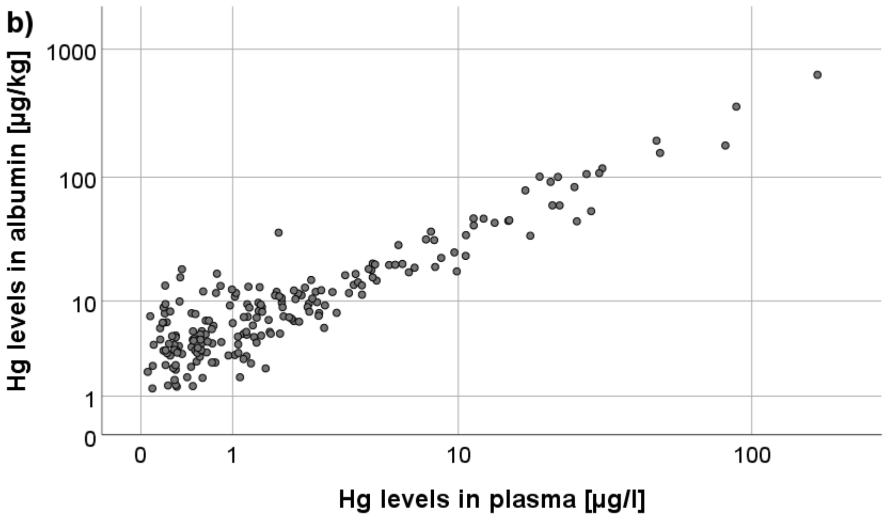

Figure S3: Box plot of the ratios of Hg levels in erythrocytes and plasma ($Hg_{E/P}$) stratified by the Exposure Risk Score (ERS).

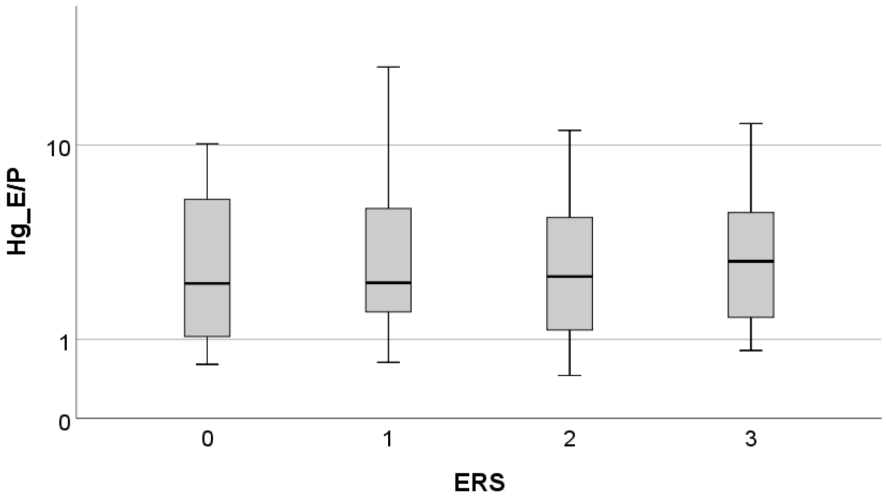

Differences between the groups were not significant (Kruskal-Wallis-Test, Jonckheere-Terpstra-Test).
